# Cooperative DNA and histone binding by Uhrf2 links the two major repressive epigenetic pathways

**DOI:** 10.1002/jcb.23185

**Published:** 2011-05-19

**Authors:** Garwin Pichler, Patricia Wolf, Christine S Schmidt, Daniela Meilinger, Katrin Schneider, Carina Frauer, Karin Fellinger, Andrea Rottach, Heinrich Leonhardt

**Affiliations:** 1 Ludwig Maximilians University Munich, Department of Biology II and Center for Integrated Protein Science Munich (CIPS^M^)Großhaderner Str. 2, 82152 Planegg-Martinsried, Germany

**Keywords:** Uhrf1, Uhrf2, DNA methylation, Histone modifications, Epigenetics

## Abstract

Gene expression is regulated by DNA as well as histone modifications but the crosstalk and mechanistic link between these epigenetic signals are still poorly understood. Here we investigate the multi-domain protein Uhrf2 that is similar to Uhrf1, an essential cofactor of maintenance DNA methylation. Binding assays demonstrate a cooperative interplay of Uhrf2 domains that induces preference for hemimethylated DNA, the substrate of maintenance methylation, and enhances binding to H3K9me3 heterochromatin marks. FRAP analyses revealed that localization and binding dynamics of Uhrf2 in vivo require an intact tandem Tudor domain and depend on H3K9 trimethylation but not on DNA methylation. Besides the cooperative DNA and histone binding that is characteristic for Uhrf2, we also found an opposite expression pattern of *uhrf1* and *uhrf2* during differentiation. While *uhrf1* is mainly expressed in pluripotent stem cells, *uhrf2* is upregulated during differentiation and highly expressed in differentiated mouse tissues. Ectopic expression of Uhrf2 in *uhrf1*^*−/−*^ embryonic stem cells did not restore DNA methylation at major satellites indicating functional differences. We propose that the cooperative interplay of Uhrf2 domains may contribute to a tighter epigenetic control of gene expression in differentiated cells.

DNA methylation and histone modifications are major epigenetic marks involved in the regulation of gene expression, inheritance of chromatin states, genome stability, and cellular differentiation [Bird, 2002; Kouzarides, 2007; Reik, 2007]. Misregulation of epigenetic pathways, like erroneous DNA methylation, may lead to cancer and other diseases [Jones and Baylin, 2007]. Open questions concern the crosstalk and mechanistic link between different epigenetic signals.

Genome-scale DNA methylation studies revealed a connection between DNA methylation and histone modifications. Specifically, DNA methylation correlates with the absence of H3K4 methylation and presence of H3K9 methylation [Meissner et al., 2008]. This correlation may in part be caused by DNA methyltransferases specifically recognizing histone modifications. For instance, the de novo DNA methyltransferase Dnmt3a and its cofactor Dnmt3L specifically recognize unmethylated H3K4 mediated by the ATRX-Dnmt3-Dnmt3L (ADD) domain [Ooi et al., 2007; Otani et al., 2009]. Dnmt1, which is involved in maintenance methylation during DNA replication and DNA repair [Leonhardt et al., 1992; Mortusewicz et al., 2005], specifically methylates hemimethylated DNA [Bestor and Ingram, 1983; Pradhan et al., 1997] and associates with constitutive heterochromatin via its targeting sequence (TS) domain [Easwaran et al., 2004].

Recently, Uhrf1 (also known as Np95 or ICBP90) has been shown to link DNA and histone modifications and has emerged as an essential cofactor for the maintenance of genomic DNA methylation. Genetic ablation of *uhrf1* leads to remarkable genomic hypomethylation, a phenotype similar to *dnmt1*^*−/−*^ embryonic stem cells (ESCs) [Bostick et al., 2007; Sharif et al., 2007]. Uhrf1 binds hemimethylated DNA via a SET and RING associated domain (SRA) domain and targets Dnmt1 to its substrate of maintenance DNA methylation [Bostick et al., 2007; Sharif et al., 2007; Arita et al., 2008; Avvakumov et al., 2008; Hashimoto et al., 2008; Qian et al., 2008; Rottach et al., 2010]. This targeting activity of Uhrf1 is based on specific binding to the heterochromatin mark H3K9me3 via a tandem Tudor domain (TTD) [Karagianni et al., 2008; Rottach et al., 2010]. In addition, Uhrf1 interacts with Dnmt3a and Dnmt3b and with histone modifying enzymes like HDAC1, G9a, and Tip60 [Unoki et al., 2004; Achour et al., 2009; Kim et al., 2009; Meilinger et al., 2009]. Finally, Uhrf1 displays E3 ubiquitin ligase activity for histone H3 [Citterio et al., 2004] and is involved in large scale reorganization of chromocenters [Papait et al., 2008].

Interestingly, a second member of the Uhrf family, Uhrf2, harbors similar domains [Bronner et al., 2007]. Until now, the only known function of Uhrf2 is a role in intranuclear degradation of polyglutamine aggregates [Iwata et al., 2009]. In this study, we systematically investigated the function and interplay of distinct Uhrf2 domains in DNA and histone tail substrate recognition and report first hints on cell-type specific functions of Uhrf1 and Uhrf2.

## MATERIALS AND METHODS

### Expression Constructs

Expression constructs for GFP, RFP-PCNA, Uhrf1-GFP, and GFP constructs of Dnmt1 were described previously [Sporbert et al., 2005; Fellinger et al., 2009; Meilinger et al., 2009]. All Uhrf2 expression constructs were derived by PCR from mouse *uhrf2*-myc cDNA (MR210744, ORIGENE). To obtain GFP fusion constructs, the *uhrf1* cDNA [Rottach et al., 2010] was replaced by *uhrf2* encoding PCR fragments in the pCAG-*uhrf1*-GFP vector. The deletion and point mutant expression constructs were derived from the corresponding wild-type constructs by overlap extension PCR [Ho et al., 1989] and PCR-based mutagenesis. The following start and end amino acids were chosen: Uhrf2 tandem Tudor domain, amino acids 118–312; Uhrf2 PHD domain, amino acids 325–395; Uhrf2 tandem Tudor–PHD domain, amino acids 118–395; Uhrf1 tandem Tudor–PHD domain, amino acids 121–370. The linker exchange constructs were derived by PCR using overlapping primers that contained the partial linker sequence. Amino acid sequences of the linkers: Uhrf1: KERRPLIASPSQPPA; Uhrf2: GAHPISFADGKF. All constructs were verified by DNA sequencing. Throughout this study enhanced GFP constructs were used and for simplicity referred to as GFP fusions.

### Cell Culture, Transfection, Cell Sorting, and Differentiation

HEK293T cells, MEFs, and ESCs were cultured and transfected as described [Schermelleh et al., 2007; Rottach et al., 2010] with the exception that Lipofectamin (Invitrogen) was used for transfection of MEFs. E14 *uhrf1*^*−/−*^ ESCs were transfected with Uhrf1-GFP and Uhrf2-GFP expression constructs using FuGENE HD (Roche) according to the manufacturer's instructions. ESCs were sorted for GFP positive cells 48 h after transfection with a FACS Aria II instrument (Becton Dikinson). ESC strains wt E14, wt J1, and E14 *uhrf1*^*−/−*^ were cultured and differentiated to embryoid bodies as described [Szwagierczak et al., 2010]. The ESC strain wt JM8A3.N1 (EUCOMM, Germany) was cultured in Knockout D-MEM (Gibco-BRL, Grand-Island, NY) medium containing 10% fetal bovine serum (PAA Laboratories GmbH, Austria), 0.1 mM β-mercaptoethanol (Gibco-BRL), 2 mM l-glutamine, 100 U/ml penicillin, 100 µg/ml streptomycin (PAA Laboratories GmbH). The medium was supplemented with 1,000 U/ml recombinant mouse LIF (Millipore, Temecula, CA).

### RNA Isolation, cDNA Synthesis, and Quantitative Real-Time PCR

RNA isolation and cDNA synthesis were performed as described [Szwagierczak et al., 2010]. Equal amounts of cDNA were used for Real-time PCR with TaqMan Gene Expression Master Mix (Applied Biosystems) on the 7500 Fast Real-time PCR System (Applied Biosystems) according to the manufacturer's instructions. The following TaqMan Gene expression assays were used: Gapdh (Assay ID: Mm99999915_g1), uhrf1 (Assay ID: Mm00477865_m1) and uhrf2 (Assay ID: Mm00520043_m1). Gene expression levels were normalized to Gapdh and calculated using the comparative C_T_ Method (ΔΔC_T_ Method).

### In Vitro DNA Binding and Histone-Tail Peptide Binding Assay

The in vitro binding assays were performed as described previously [Frauer and Leonhardt, 2009; Rottach et al., 2010]. NoCpG DNA substrates were produced in a primer extension reaction [Frauer and Leonhardt, 2009] others by hybridization of two DNA oligos (Supplementary Fig. S7B–D). Histone-tail peptides were purchased as TAMRA conjugates (PSL, Germany; Supplementary Fig. S7A). Peptides were added in a molar ratio 1.5:1 (peptide/GFP fusion) and the binding reaction was performed at RT for 15 min with constant mixing. For combined assays, samples were additionally incubated with either H3K9me3 or H3K9ac histone-tail peptides in a molar ratio 1.5:1 (peptide/GFP fusion) or increasing amount of DNA substrate as indicated. The binding reaction was performed at RT for 60 min with constant mixing.

### Immunoflourescence Staining and Antibodies

For immunostaining, MEF cells and ESCs were grown on cover slips and transiently transfected with Uhrf2-GFP (MEF cells), or co-transfected with Uhrf2-GFP and RFP-PCNA (ESCs). Cells were fixed with 2.0% or 3.7% formaldehyde in PBS and permeabilized in PBS containing 0.2% Triton X-100. The post-translational histone modification H3K9me3 was detected via a rabbit primary antibody (Active Motif) and a secondary anti-rabbit antibody conjugated to Alexa Fluor 594 (Molecular Probes, Eugene, OR). The antibodies were diluted 1:1,000 or 1:500, respectively, in PBS containing 0.02% Tween-20 and 2% BSA. GFP-Binder (ChromoTek, Germany) was used to boost GFP signals and was labeled with Alexa Fluor 488. Cells were counterstained with DAPI and mounted in Vectashield (Vector Laboratories, Burlingame, CA). Images of the cells were obtained using a TCS SP5 AOBS confocal laser scanning microscope (Leica, Wetzlar, Germany) with a 63x/1.4 NA Plan-Apochromat oil immersion objective. GFP, Alexa Fluor 488, RFP, and Alexa Fluor 594 were excited with a 488-nm argon laser and a 561-nm diode laser, respectively. Image series were recorded with a frame size of 512 × 512 pixels, a pixel size of 100 nm and with a detection pinhole size of 1 Airy Unit.

### Live Cell Microscopy and Fluorescence Recovery After Photobleaching (FRAP) Analysis

Live cell imaging and FRAP analyses were performed as described [Schermelleh et al., 2007] with the exception that imported images were intensity normalized, converted to 8-bit and Gauss-filtered (2 pixel radius). Data sets showing lateral movement were corrected by image registration using the StackReg plug-in of ImageJ [Abramoff et al., 2004] starting with a frame when approximately half recovery was reached. Within the first 30 s after bleaching, images were taken every 150 ms and then in intervals of 1 s.

### DNA Methylation Analysis

Genomic DNA was isolated with the QIAmp DNA Mini Kit (Qiagen) and 1.5 µg were bisulfite converted using the EZ DNA Methylation-Gold Kit (Zymo research) according to the manufacturer's instructions. Primer sequences for major satellites were AAAATGAGAAACATCCACTTG (forward primer) and CCATGATTTTCAGTTTTCTT (reverse primer). For amplification we used Qiagen Hot Start Polymerase in 1× Qiagen Hot Start Polymerase buffer supplemented with 0.2 mM dNTPs, 0.2 µM forward primer, 0.2 µM reverse primer, 1.3 mM betaine (Sigma) and 60 mM tetramethylammonium-chloride (TMAC, Sigma). Major satellites were amplified in a single amplification and pyrosequencing reactions were carried out by Varionostic GmbH (Ulm, Germany).

### Statistical Analysis

Results were expressed as means ± SD or means ± SEM. The difference between two mean values was analyzed by Student's *t*-test and was considered as statistically significant in case of *P* < 0.05 (*) and highly significant for *P* < 0.001 (**).

## RESULTS

### Opposite Expression Pattern of *uhrf1* and *uhrf2* During Differentiation

**Figure 1:**
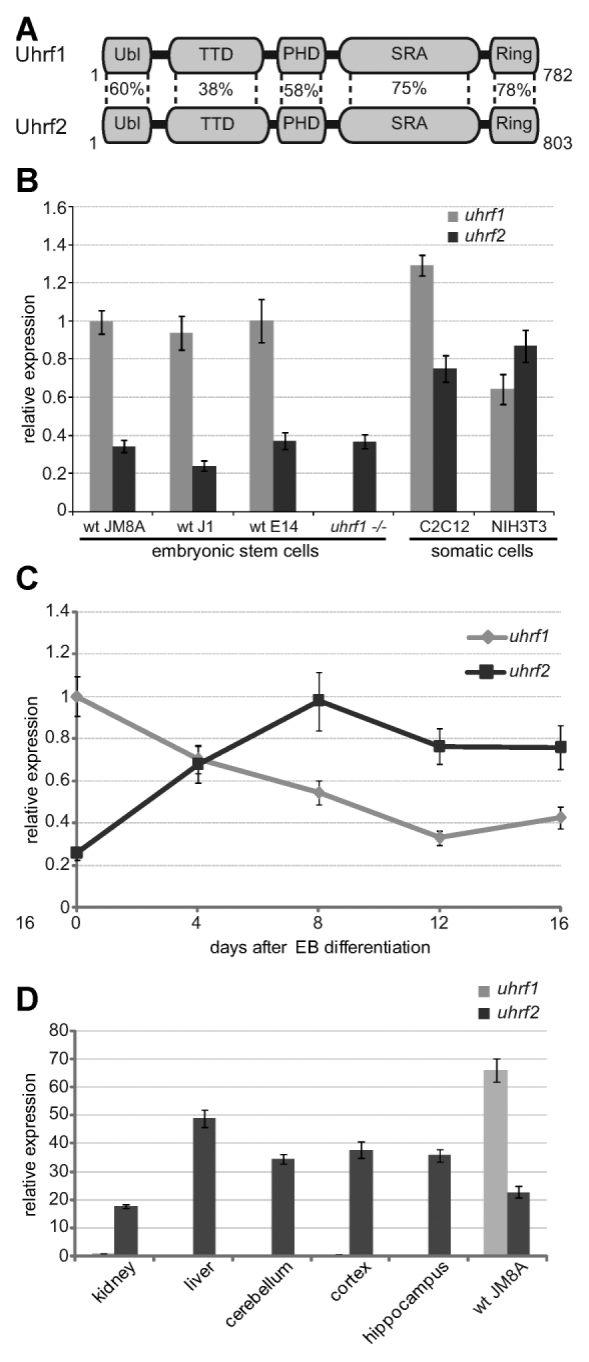
Opposite expression pattern of *uhrf1* and *uhrf2* during differentiation. A: Schematic outline of the multi-domain architecture of Uhrf1 in comparison to Uhrf2. An N-terminal ubiquitin-like domain (Ubl) is followed by a tandem Tudor domain (TTD), a plant homeodomain (PHD), a SET and RING associated (SRA) domain and a C-terminal really interesting new gene (RING) domain. Numbers indicate primary sequence similarities of single domains determined by BlastP search [Altschul, 1991]. Expression analysis of *uhrf1* and *uhrf2* by Real-time PCR in ESCs and somatic cells (B), during differentiation of wt J1 ESCs (C) and in various adult mouse tissues in comparison to the expression data in ESCs (D). Expression levels are relative to *uhrf1* in wtJM8A (B), day 0 of differentiation (C) and to kidney (D) (*uhrf1* set to 1). Shown are means ± SD of at least two independent experiments.

Recently, Uhrf1 has emerged as an essential factor for the maintenance of DNA methylation. Sequence analyses revealed that Uhrf2 harbors five recognizable domains similar to Uhrf1 (Fig. 1A), but its role in the regulation of DNA methylation is still unclear. We compared the expression pattern of *uhrf1* and *uhrf2* in ESCs and somatic cells, during differentiation and in differentiated mouse tissues (Fig. 1B–D and Supplementary Fig. S1). Interestingly, both genes show opposite expression patterns; while *uhrf1* is expressed in ESCs and down regulated during differentiation, which is consistent with previous reports [Muto et al., 1995; Fujimori et al., 1998; Hopfner et al., 2000], *uhrf2* is upregulated and highly expressed in differentiated mouse tissues. The switch in the expression pattern argues against a functional redundancy of both genes and is consistent with the drastic loss of DNA methylation in *uhrf1*^*−/−*^ ESCs despite the presence of intact *uhrf2* alleles. Therefore, the opposite expression pattern of both genes suggests different functional roles of *uhrf1* and *uhrf2* in development.

### Cooperative Binding of Repressive Epigenetic Marks by Uhrf2

**Figure 2:**
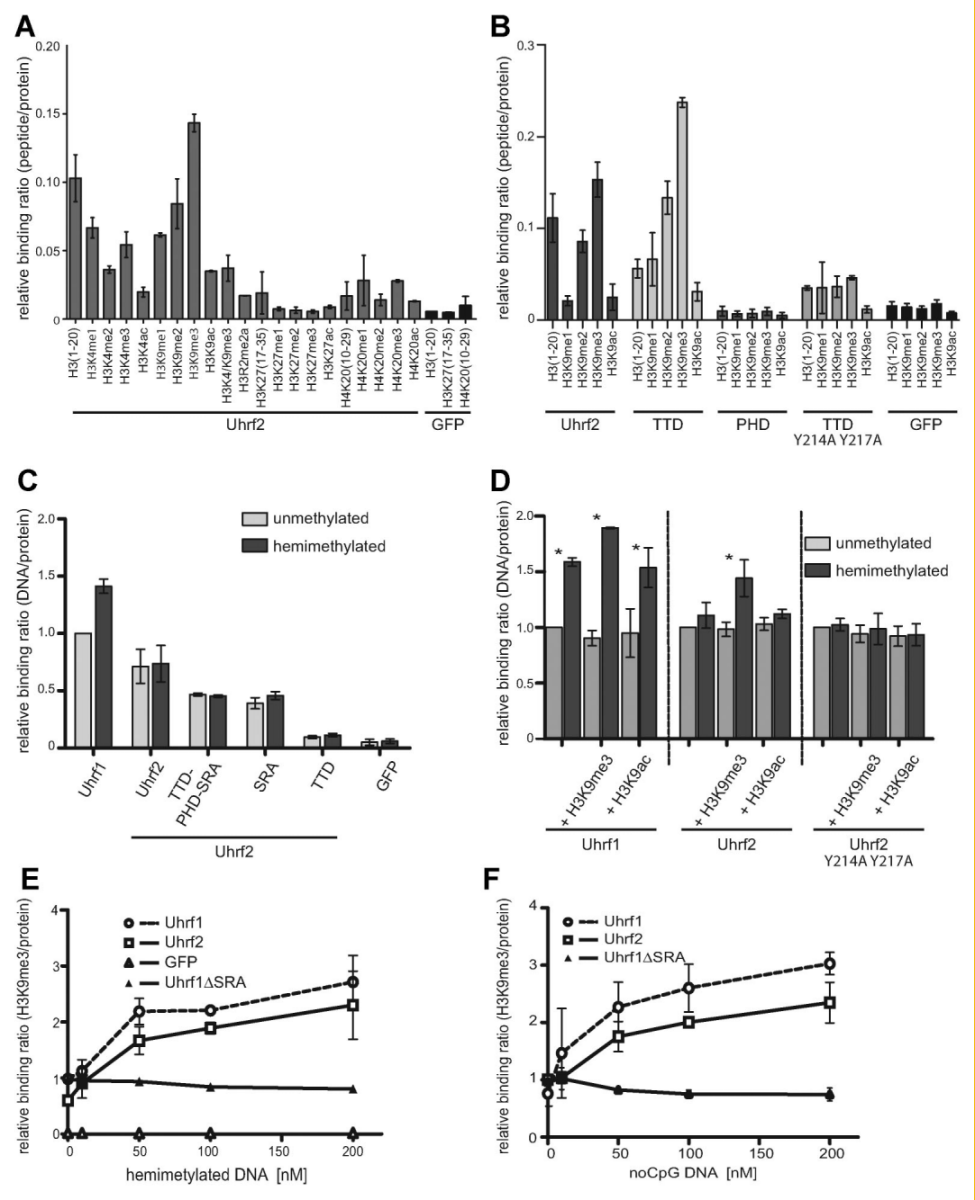
Cooperative binding of repressive epigenetic marks by Uhrf2. In vitro binding ratios of fluorescently labeled substrate over bound GFP fusion proteins were determined. A: Histone H3- and H4-tail binding specificities of Uhrf2. Shown are means ± SD of biological duplicates. B: Histone H3 tail binding specificity of Uhrf2, its tandem Tudor domain (TTD), its PHD domain and its TTD mutant (Y214A Y217A). Shown are means ± SEM of at least three independent experiments. C: DNA binding properties of Uhrf1, Uhrf2 and of single (SRA, TTD) and combined Uhrf2 domains (TTD–PHD–SRA). Shown are means ± SEM of three independent experiments. D: DNA binding properties of Uhrf1, Uhrf2 and Uhrf2 Y214A Y217A in combination with histone-tail peptide binding. Shown are means ± SD of three independent experiments (Uhrf1, Uhrf2) and of two independent experiments (Uhrf2 Y214A Y217A). Values were normalized to the binding ratio of each GFP fusion for unmethylated DNA without histone-tail peptide. Statistical significance of differences between the binding ratios with un- and hemimethylated DNA is indicated; *P < 0.05. E + F: H3K9me3 peptide binding by Uhrf1, Uhrf2, and Uhrf1ΔSRA with increasing concentrations of DNA substrate containing either one central hemimethylated (E) or noCpG site (F). Shown are means ± SD of biological duplicates. Values were normalized to the binding ratio of Uhrf1ΔSRA without DNA.

To investigate DNA and histone-tail binding preferences of Uhrf2 in vitro, we used a versatile binding assay developed for GFP fusion proteins [Rothbauer et al., 2008; Frauer and Leonhardt, 2009; Rottach et al., 2010]. Similar to Uhrf1, histone-tail peptide binding assays revealed that Uhrf2 preferentially binds to H3(1–20) and H3K9me3 peptides (Fig. 2A). This binding activity of Uhrf2 is mediated by the TTD but not the PHD domain (Fig. 2B). Consistently, acetylation of H3K9, underrepresented in heterochromatin, prevented the binding of Uhrf2 and its TTD. The binding of Uhrf1 to H3K9me3 is mediated by an aromatic cage in the TTD [Rottach et al., 2010]. Site-directed mutagenesis of Uhrf2 changing the two conserved tyrosine residues to alanine (Y214A Y217A) (Supplementary Fig. S2) abolished specific peptide binding (Fig. 2B) and supports a function of the aromatic cage in H3K9me3 recognition.

Whereas Uhrf1 preferentially binds to hemimethylated DNA, Uhrf2 failed to show a preference for hemi-over unmethylated DNA (Fig. 2C). These differences in DNA binding preferences between Uhrf1 and Uhrf2 were confirmed by electrophoretic mobility shifts (Supplementary Fig. S3). To further investigate the functional interplay between DNA and histone binding we performed combined binding assays (Fig. 2D). Interestingly, binding to heterochromatin-specific H3K9me3 peptides induced a significant preference of Uhrf2 for hemi-over unmethylated DNA. Uhrf1 already on its own showed preference for hemimethylated DNA that was further enhanced by binding to H3K9me3 peptides. To test the specificity of this cooperativity we mutated the aromatic cage in Uhrf2 that is necessary for H3K9me3 histone-tail peptide binding. The mutated Uhrf2 (Y214A Y217A) showed comparable DNA binding activity as the wild-type Uhrf2 but addition of heterochromatin-specific H3K9me3 peptides did not induce preference for hemi-over unmethylated DNA (Fig. 2D).

In the reverse experiment, addition of DNA enhanced binding of Uhrf1 and Uhrf2 to the H3K9me3 peptide (Fig. 2E,F). This was not observed for the DNA binding mutant of Uhrf1 (Uhrf1ΔSRA) which showed constant peptide binding with increasing DNA concentrations. These findings suggest that single binding events of distinct Uhrf2 domains lead to multivalent engagement of different repressive epigenetic marks. In fact, multivalent engagement of DNA and histone tail peptides via the SRA domain and the TTD, respectively, results in affinity enhancement and additional specificity for hemimethylated DNA, the substrate of maintenance methylation.

### Cellular Localization and Dynamics of Uhrf2 Depend on Histone H3K9 Methylation

**Figure 3:**
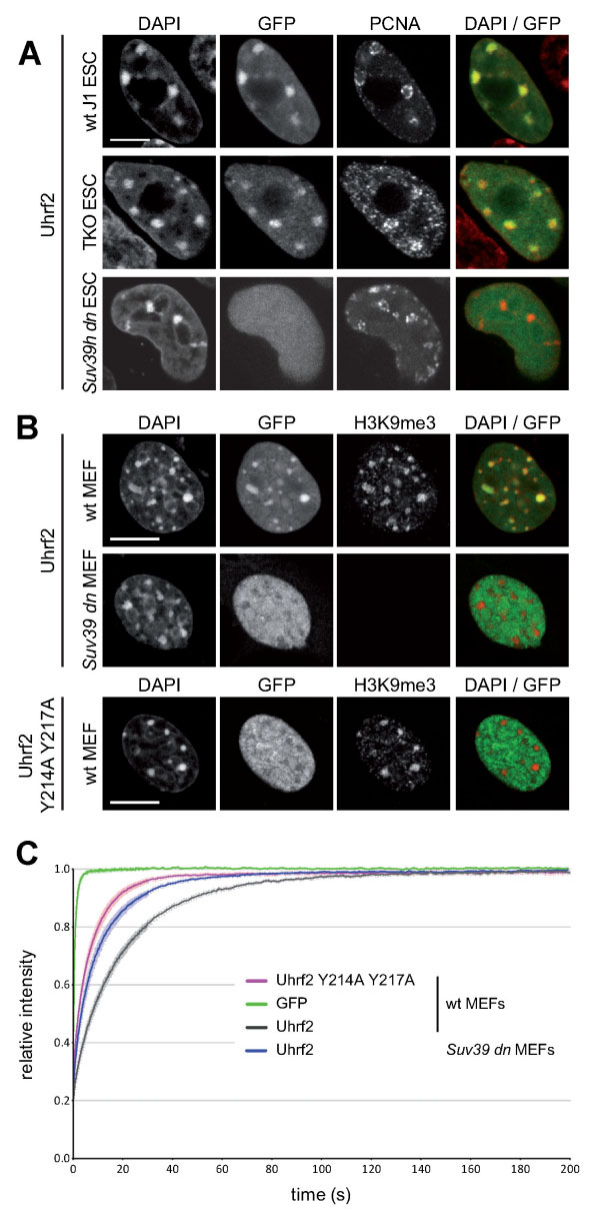
Cellular localization and dynamics of Uhrf2 depend on histone H3K9 methylation. A: Confocal mid sections of fixed wt J1, TKO and *Suv39h dn* ESCs transiently expressing Uhrf2-GFP and RFP-PCNA and counterstained with DAPI, which preferentially highlights PH. Merged images are displayed on the right side (GFP: green; DAPI: red). Scale bar 5 µm. B: Confocal mid sections of fixed wt MEFs and *Suv39h dn* MEFs transiently expressing Uhrf2-GFP or Uhrf2 Y214A Y217A-GFP were immunostained for H3K9me3 and counterstained with DAPI. Merged images are displayed on the right side (GFP: green; DAPI: red). Scale bar 5 µm. C: Dynamics of Uhrf2-GFP and Uhrf2 Y214A Y217A-GFP in living MEFs determined by half nucleus FRAP analysis. GFP is shown as reference. Curves represent means ± SEM from at least 8 nuclei.

To monitor the subcellular localization of Uhrf2, we expressed Uhrf2-GFP constructs in cells with different genetic backgrounds. In wild type (wt) ESCs, Uhrf2 is localized in the nucleus and is enriched at pericentric heterochromatin (PH) (Fig. 3A,B and Supplementary Fig. S4A–C). To investigate which epigenetic marks at PH are recognized by Uhrf2 we determined the localization of Uhrf2 in genetically modified ESCs either lacking all three major DNA methyltransferases Dnmt1, Dnmt3a, and Dnmt3b (TKO) [Tsumura et al., 2006] or ESCs lacking the two major H3K9 methyltransferases Suv39H1/H2 (*Suv39h dn*) [Lehnertz et al., 2003]. TKO cells are practically devoid of genomic DNA methylation and *Suv39h dn* ESCs show substantially reduced H3K9me3 levels. We found Uhrf2 localized at PH in TKO but not in *Suv39h dn* ESCs, indicating that localization of Uhrf2 is dependent on H3K9 but not on DNA methylation (Fig. 3A). Consistently, immunostaining of wt mouse embryonic fibroblasts (MEFs) showed co-localization of Uhrf2 and H3K9me3 marks at PH, which was not observed in *Suv39h dn* MEFs [Peters et al., 2001] (Fig. 3B). Also, mutations in the TTD (Uhrf2 Y214A Y217A) that abolished binding to H3K9me3 peptides in vitro disrupted enrichment at PH in wt MEFs (Fig. 3B). The dependence of Uhrf2 localization on H3K9me3 was also confirmed by quantitative correlation analysis (Supplementary Fig. S4D,E).

To investigate the effect of H3K9me3 on the dynamics of Uhrf2 in living cells we performed quantitative fluorescence recovery after photobleaching (FRAP) analyses in wt and *Suv39h dn* MEFs. We chose to bleach half nuclei to include a representative number of interactions from different nuclear domains and structures in the bleached area [Rottach et al., 2010]. Recovery of Uhrf2-GFP fluorescence in *Suv39h dn* MEFs (half-time t_1/2_ = 5.9 ± 0.6 s) and of the TTD mutant in wt MEFs (t_1/2_ = 3.2 ± 0.4 s) was considerably faster than the recovery of Uhrf2-GFP in wt MEFs (t_1/2_ = 11.8 ± 0.6 s) pointing to a crucial role of H3K9me3 in Uhrf2 dynamics in living cells (Fig. 3C). Taken together, these results clearly demonstrate that the interaction of Uhrf2 with the heterochromatin mark H3K9me3 is required for the localization at PH and affects binding dynamics in living cells.

### Cooperative Binding of the Combined Uhrf2 TTD–PHD Domain

**Figure 4:**
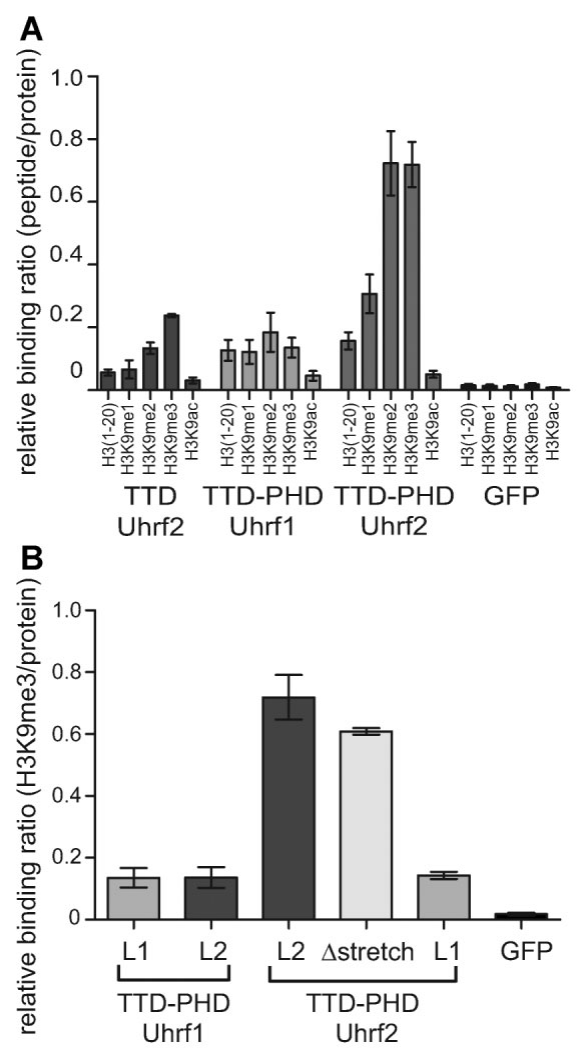
Cooperative binding of the combined tandem Tudor–PHD domain of Uhrf2. A: Histone H3 N-terminal tail binding specificity of the TTD of Uhrf2 and of the combined TTD and PHD domain (TTD–PHD) of Uhrf1 and Uhrf2. Shown are means ± SEM from at least six independent experiments. B: Histone H3K9me3 binding of the combined TTD–PHD domains of Uhrf1 and Uhrf2, hybrid proteins (L1 and L2 specify inserted linker sequences derived from Uhrf1 and Uhrf2, respectively) and a stretch deletion Uhrf2 construct. Shown are means ± SEM from at least three independent experiments.

Recently, several studies showed multivalent binding to histone-tail peptides [Ruthenburg et al., 2007]. In case of Uhrf1 and Uhrf2, the TTD is followed by a second histone-tail binding domain, a PHD domain (Fig. 1A). As the isolated PHD domains of Uhrf1 and Uhrf2 did not show binding to H3 histone-tail peptides (Fig. 2B) [Rottach et al., 2010], we tested whether the combination of the PHD and the TTD results in cooperative histone-tail binding. Surprisingly, the combined TTD–PHD domain of Uhrf2 displayed a fourfold increased binding to H3K9me2/me3 in comparison to the single TTD, which was not observed for the corresponding construct of Uhrf1 (Figs. .2B and 4A).

Sequence alignments of the combined domains revealed two striking differences between Uhrf1 and Uhrf2. Firstly, Uhrf2 harbors an additional stretch of 33 highly conserved amino acids present in the TTD (Supplementary Fig. S5A). Secondly, the linker region between the TTD and PHD domain of Uhrf2 is highly conserved, whereas this region is highly diverse in Uhrf1 (Supplementary Fig. S5A). To test which sequence is responsible for the observed cooperative interplay between PHD and TTD, we generated and tested different hybrid and deletion constructs (Supplementary Fig. S5B). Notably, replacement of the native linker in the Uhrf2 TTD–PHD construct by the Uhrf1 linker caused decreased relative binding ratios to H3K9me2/3 comparable to the single Uhrf2 TTD (Fig. 4B). Transferring the Uhrf2 linker to the Uhrf1 TTD–PHD construct as well as deletion of the Uhrf2 stretch region did not affect the binding to H3K9me3 peptides (Fig. 4B).

These results suggest that the cooperative interplay of different Uhrf2 domains, which is responsible for the increased binding to heterochromatin marks, is dependent on the highly conserved linker region connecting the TTD and PHD domains. A similar functional importance of linker sequences has been described for BPTF and histone lysine demethylases [Li et al., 2006; Horton et al., 2010].

### Uhrf1 and Uhrf2 Are Not Functionally Redundant in ESCs

**Figure 5:**
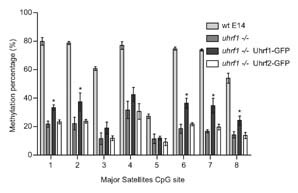
Uhrf1 and Uhrf2 are not functionally redundant in ESCs. DNA methylation analysis of wt E14 ESCs, *uhrf1^−/−^* ESCs and of *uhrf1^−/−^* ESCs ectopically expressing Uhrf1-GFP or Uhrf2-GFP. ESCs transiently expressing Uhrf1-GFP and Uhrf2-GFP were isolated by FACS sorting 48 h after transfection and CpG methylation levels of major satellites repeats were analysed by bisulfite treatment, PCR amplification and direct pyrosequencing. Statistical significance of differences in DNA methylation levels between *uhrf1^−/−^* ESCs and *uhrf1^−/−^* ESCs with ectopically expressed Uhrf1-GFP or Uhrf2-GFP are indicated; *P < 0.05. Shown are means ± SD from three independent experiments.

To investigate whether Uhrf1 and Uhrf2 are functionally redundant we performed interaction and rescue assays. Like Uhrf1, also Uhrf2 interacts with Dnmts (Supplementary Fig. S6) suggesting a similar function in DNA methylation. To test for such a functional role, we ectopically expressed Uhrf2-GFP or Uhrf1-GFP in *uhrf1*^*−/−*^ ESCs and determined DNA methylation levels at major satellites by pyrosequencing. While ectopic expression of Uhrf1-GFP led to significant increase of DNA methylation levels at CpG sites of major satellite DNA in *uhrf1*^*−/−*^ ESCs, Uhrf2-GFP did not restore DNA methylation at these sites (Fig. 5). These results point to functional differences between Uhrf1 and Uhrf2 in vivo.

## DISCUSSION

Over the past decades many different histone modifications were discovered that are involved in epigenetic gene regulation. A key question is how these histone marks are linked to DNA methylation pattern and how this complex epigenetic information is integrated and translated into defined chromatin structures and gene expression levels. Epigenetic regulators that bind DNA and histone marks are ideally suited to link these pathways and intramolecular interactions between different binding domains may contribute to substrate specificity and epigenetic regulation [Hashimoto et al., 2009].

Recently, Uhrf1, an essential factor for the maintenance of DNA methylation, has been shown to bind to repressive DNA and histone modifications via an SRA and a tandem Tudor domain, respectively. Here we provide the first systematic characterization of the second member of the Uhrf family, Uhrf2, and demonstrate that Uhrf2 binds to the H3K9me3 heterochromatin mark via an aromatic cage of a tandem Tudor domain (TTD). Mutations in the aromatic cage abolished binding to H3K9me3 histone-tail peptides in vitro and prevented enrichment of Uhrf2 at pericentric heterochromatin in vivo. Interestingly, similar mutations in the aromatic cage of Uhrf1 prevented repression of *p16*^*INK4A*^ [Nady et al., 2011] suggesting a link between H3K9me3 binding and a function of Uhrf proteins in gene repression.

Our results point to a complex regulation of substrate recognition by Uhrf2 involving cooperative binding domains and critical linker sequences. In contrast to Uhrf1, preferential binding of Uhrf2 to hemimethylated DNA, the substrate of DNA maintenance methylation, was only induced upon simultaneous binding to H3K9me3 histone-tail peptides. Binding of Uhrf1 and Uhrf2 to DNA in turn enhanced binding to H3K9me3 histone-tail peptides. Consistently, SILAC-based proteomic analysis identified enrichment of UHRF1 at nucleosomes containing repressive DNA and H3K9 methylation marks [Bartke et al., 2010]. Together, these data demonstrate a cooperative interplay between DNA and histone tail binding domains of Uhrf1 and Uhrf2. A similar effect was reported for MSL3 that specifically binds to H4K20me1 via a chromodomain only in the presence of DNA [Kim et al., 2010].

An additional level of complexity was added by recent studies showing multivalent binding of histone-tail peptides by mixed two-effector modules [Ruthenburg et al., 2007]. Notably, the combined TTD–PHD domain of Uhrf2, but not of Uhrf1, showed enhanced binding to H3K9me3 histone-tail peptides. This cooperativity was dependent on the highly conserved linker region connecting the TTD and PHD domains. Similarly, an important role was attributed to the linker sequence between the histone binding domain (PHD) and the histone modifying domain of jumanji histone lysine demethylases [Horton et al., 2010].

The dramatic loss of DNA methylation in *uhrf1*^*−/−*^ ESCs [Bostick et al., 2007; Sharif et al., 2007] is remarkable, especially considering the presence of the *uhrf2* gene, which encodes a highly similar protein as demonstrated in this study. As one possible explanation for this lack of functional redundancy we found, in contrast to *uhrf1*, relatively low *uhrf2* mRNA levels in ESCs, which were not affected by genetic *uhrf1* ablation. Moreover, both genes also show opposite expression patterns during differentiation. The failure of ectopically expressed Uhrf2 to restore DNA methylation in *uhrf1* deficient cells clearly points to functional differences between both proteins in vivo. However, more definitive insights into the specific function(s) of Uhrf2 will require targeted mutations and subsequent analyses of pluripotent as well as differentiated cells. Based on the cooperative binding of Uhrf2 domains to repressive DNA and histone marks we propose that Uhrf2 might contribute to a tighter control of gene repression in differentiated cells as compared to a less stringent control by Uhrf1 in pluripotent ESCs.
