# Supplementary material for: Cooperative DNA and histone binding by Uhrf2 links the two major repressive epigenetic pathways
Source: J Cell Biochem. 2011 May 19;112(9):2585–93. doi: 10.1002/jcb.23185 (PMC3569875; doi:10.1002/jcb.23185)
Supplement: Supplementary file 1 [file jcb0112-2585-sd1.pdf]

Supplementary Information

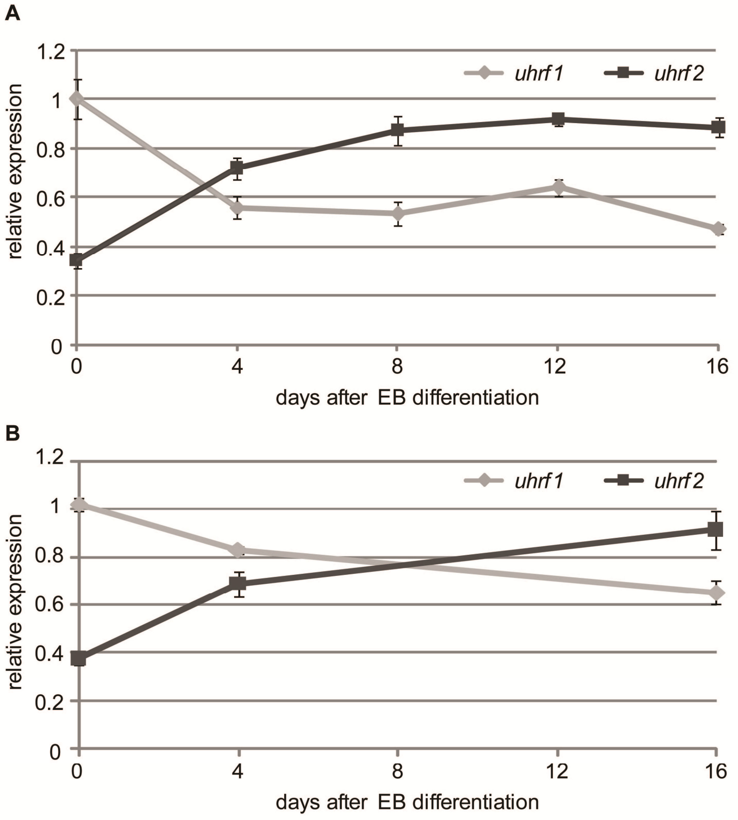

Supplementary Figure S1. Opposite expression pattern of *uhrf1* and *uhrf2*. Expression analyses of *uhrf1* and *uhrf2* by Real-time PCR during differentiation of ESCs with two different genetic backgrounds (wt E14 (**A**) and wt JM8A (**B**)). Transcript levels of *uhrf1* at day 0 of EB formation are used as reference point (set to 1). Shown are means ± SD from three technical replicates of one biological experiment.

A
B

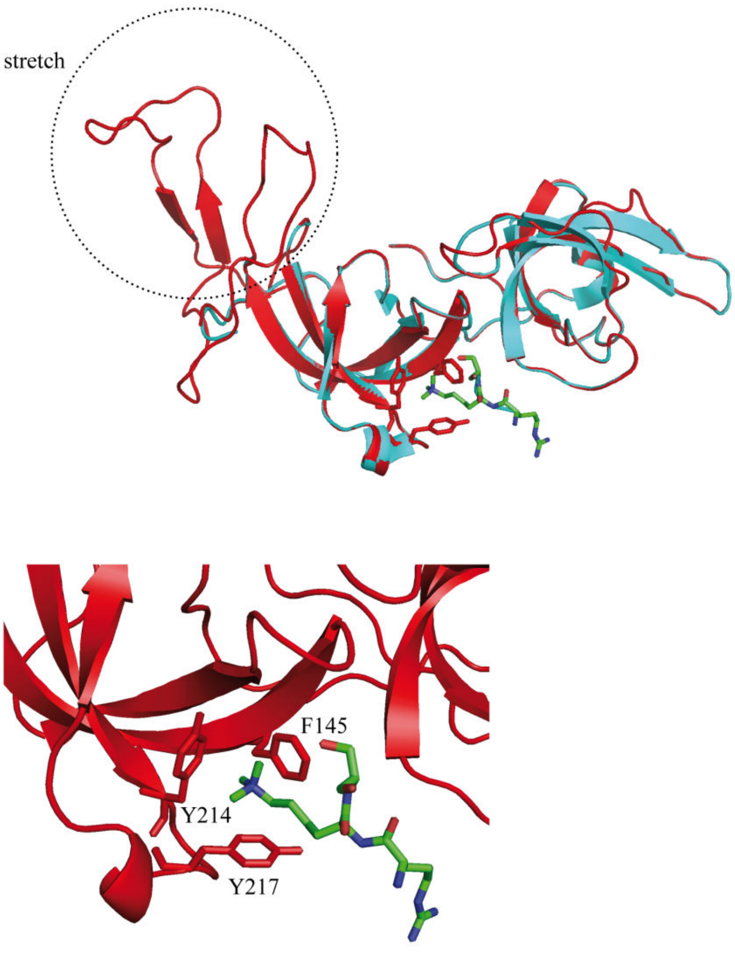

Supplementary Figure S2. Model of the tandem Tudor domain (TTD) of Uhrf2. (**A**) A model of the TTD of Uhrf2 was generated using SWISS Model [Arnold et al., 2006; Guex and Peitsch, 1997] with the solved structure of the TTD of Uhrf1 (PDB: 3DB3) as template. Both structures, the Uhrf2 model in red and the Uhrf1 template in cyan, are superimposed in PyMOL [Schrodinger, 2010]. (**B**) H3K9me3 is embedded in an aromatic cage formed by three aromatic residues of Uhrf2.

A

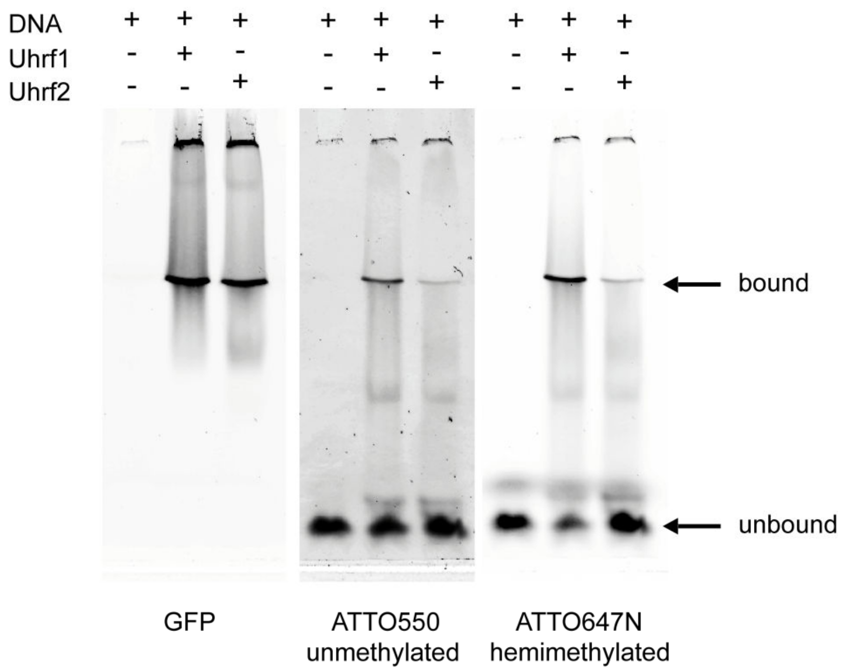

B

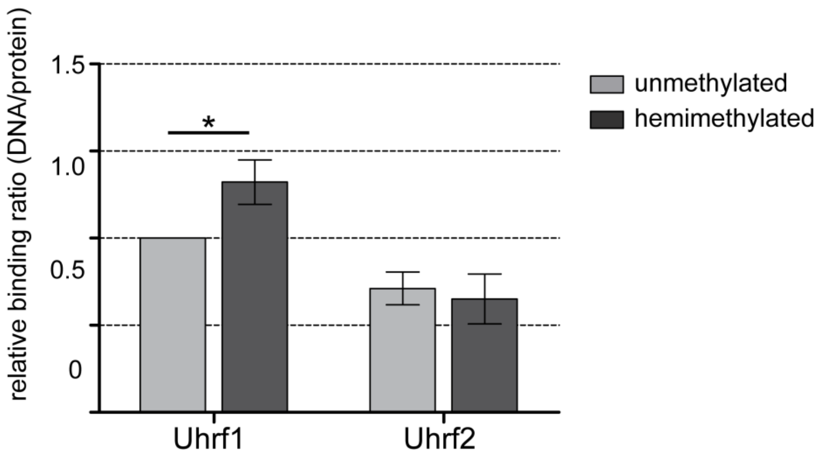

Supplementary Figure S3. Electrophoretic mobility shift of Uhrf1 and Uhrf2. (**A**) Un- and hemimethylated DNA substrates (1 pmol each in direct competition) were incubated with 0.63 pmol purified Uhrf1-GFP or Uhrf2-GFP. Samples were subjected to 3.5% non-denaturing PAGE and analyzed with a fluorescence scanner (Typhoon TRIO scanner, GE Healthcare) to detect ATTO550 (unmethylated substrate), ATTO647N (hemimethylated substrate) and GFP. (**B**) Band intensities were quantified with ImageJ [Abramoff, 2004]. To quantify bound DNA/protein ratios, grey values of unbound DNA bands were subtracted from the corresponding DNA input bands and subsequently normalized by the grey values of the GFP bands. All values were normalized to the relative binding ratio of Uhrf1 to unmethylated substrate. Shown are means ± SD from three independent experiments. Statistical significance between the binding ratios of un- and hemimethylated DNA is indicated; $^*P < 0.05$.

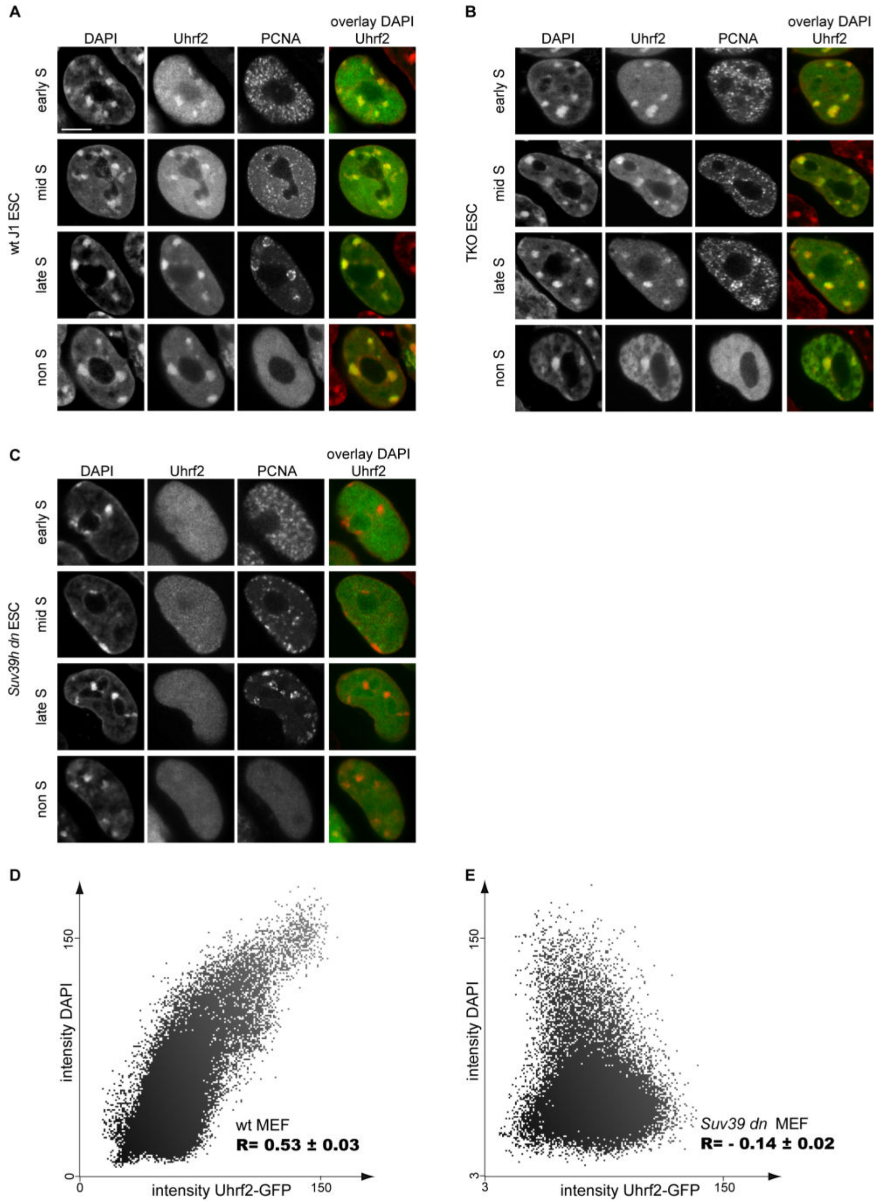

Supplementary Figure S4. Cell-cycle dependent localization of Uhrf2 in cells with different genetic backgrounds. Confocal mid sections of fixed wt J1 (**A**), TKO (**B**) and *Suv39h dn* ESCs (**C**), transiently expressing Uhrf2-GFP. Cells were co-transfected with a RFP-PCNA expression vector to distinguish S phase stages [Sporbert et al., 2005] and counterstained with DAPI. Merged images are displayed on the right. Scale bar 5 µm. In wt J1 and TKO ESCs the Uhrf2 fusion protein accumulates at pericentric heterochromatin independent of the cell-cycle stage and methylation levels (**A**) (**B**). In contrast, Uhrf2-GFP shows a fully dispersed nuclear distribution in *Suv39h dn* cells indicating the dependency on H3K9me3 methylation for localization at PH *in vivo* (**C**). (**D**) and (**E**) Scatter blot of GFP-Uhrf2 and DAPI signals in wt MEFs and *Suv39h dn* MEFs. The corresponding Pearson correlation coefficients R ± SEM are calculated from ten analysed cells. The software Volocity (Perkin Elmer) was used for analysis, selecting the cell nucleus as region of interest. Note that Pearson correlation coefficients range from +1 to -1 for perfect to no co-localization.

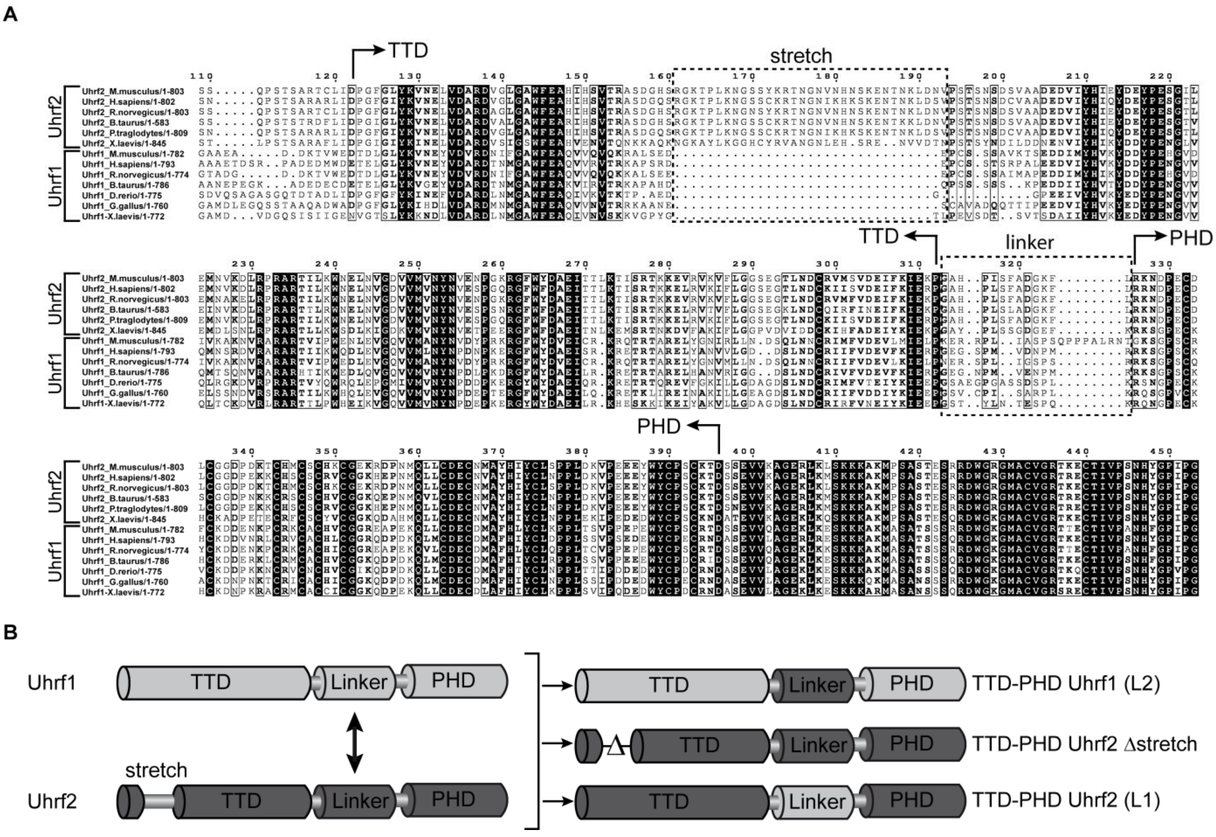

Supplementary Figure S5. Alignment and recombination of Uhrf1 and Uhrf2 domains. (**A**) Alignment of the tandem Tudor domain (TTD) and PHD domains from vertebrate Uhrf2 and Uhrf1 orthologs. Accession numbers for Uhrf2: *Homo sapiens* CAH74119.1; *Bos taurus* AAI48950.1; *Mus musculus* Q7TMI3; *Rattus norvegicus* NP_001101055.1; *Pan troglodytes* XP_528534.2; *Xenopus laevis* AAI28674.1. Accession numbers for Uhrf1: *Homo sapiens* Q96T88.1; *Bos taurus* AAI51672.1; *Mus musculus* Q8VDF2.2; *Rattus norvegicus* Q7TPK1.2; *Dario rerio* NP_998242.1; *Xenopus laevis* AAI28674.1, *Gallus gallus* XP_418269.2. Arrows show the start and end positions of the TTD and PHD domains. Absolutely conserved residues are black shaded, while positions showing conservative substitutions are boxed with residues in bold face. The additional stretch region found in the TTD of Uhrf2 and the linker region between TTD and PHD finger are boxed with dotted black lines. (**B**) Schematic outline of engineered constructs including the deletion of the stretch region and the swapping of linker sequences.

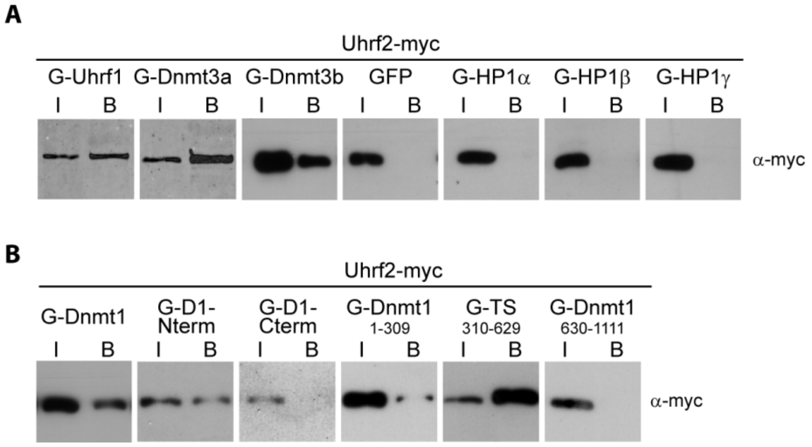

Supplementary Figure S6. Uhrf2 interacts with Uhrf1, Dnmt1 and Dnmt3a/b. (**A**) Co-immunoprecipation of Uhrf2-myc and GFP-Uhrf1, GFP-Dnmt3a, GFP-Dnmt3b, GFP-HP1α, GFP-HP1β, GFP-HP1γ or GFP transiently co-expressed in HEK293T cells. Note that Uhrf2 interacts with Uhrf1, Dnmt3a and Dnmt3b. (**B**) Co-immunoprecipation of Uhrf2-myc and GFP-Dnmt1 constructs transiently co-expressed in HEK293T cells: GFP-Dnmt1 (G-Dnmt1), GFP-fusions of the N-terminal and C-terminal part of Dnmt1 (G-D1-Nterm, G-D1-Cterm) and truncated Dnmt1 constructs (G-Dnmt1 1-309, G-TS 310-629, G-Dnmt1 630-1111). Note that Uhrf2 interacts with full-length Dnmt1, the N-terminal part and the targeting sequence (G-TS 310-629). One percent of input (I) relative to bound fractions (B) was loaded. Co-immunoprecipitation was performed using the GFP trap [Rothbauer et al., 2008]. Co-precipitated myc-tagged proteins were detected using a mouse monoclonal primary anti-myc antibody (Invitrogen, Germany) and an HRP- or Cy5-conjugated secondary anti-mouse antibody (Sigma, Germany, or Jackson ImmunoResearch Laboratories, USA, respectively).

A

Peptide name	Peptide sequence	Peptide labelling
H3K4me1	ART X1 QTARKSTGGKAPRKQLK	TAMRA at C-terminus
H3K4me2	ART X2 QTARKSTGGKAPRKQLK	
H3K4me3	ART X3 QTARKSTGGKAPRKQLK	
H3K4ac	ART Z QTARKSTGGKAPRKQLK	
H3K4/9un	ARTKQTARKSTGGKAPRKQLK	
H3K9me1	ARTKQTAR X1 STGGKAPRKQLK	
H3K9me2	ARTKQTAR X2 STGGKAPRKQLK	
H3K9me3	ARTKQTAR X3 STGGKAPRKQLK	
H3K9ac	ARTKQTAR Z STGGKAPRKQLK	
H3R2me2a	A X4 TKQTARSTGGKAPRKQLK	
H3K4me3K9me3	ART X3 QTAR X3 STGGKAPRKQLK	
H3K27un	RKQLATKAARKSAPATGGVK	TAMRA at N-terminus
H3K27me1	RKQLATKAAR X1 SAPATGGVK	
H3K27me2	RKQLATKAAR X2 SAPATGGVK	
H3K27me3	RKQLATKAAR X3 SAPATGGVK	
H3K27ac	RKQLATKAAR Z SAPATGGVK	
H4K20un	LGKGGAKRHRKVLRDNIQGI	
H4K20me1	LGKGGAKRHR X1 VLRDNIQGI	
H4K20me2	LGKGGAKRHR X2 VLRDNIQGI	
H4K20me3	LGKGGAKRHR X3 VLRDNIQGI	
H4K20ac	LGKGGAKRHR Z VLRDNIQGI	

X1: Lysine(me1); X2: Lysine(me2); X3: Lysine(me3); X4: Arginine(me2 asymmetric) Z: Lysine(ac)

B

DNA substrate	DNA sequence	DNA labelling
CGup	CTCAACAACTAACTACCATCCGGACCAGAAGAGTCATCATGG	no
MGup	CTCAACAACTAACTACCATCMGGACCAGAAGAGTCATCATGG	no
noCpG	CTCAACAACTAACTACCATCCTGACCAGAAGAGTCATCATGG	no
um647N	CCATGATGACTCTTCTGGTCCGGATGGTAGTTAGTTGTTGAG	ATTO647N at 5´end
um700	CCATGATGACTCTTCTGGTCCGGATGGTAGTTAGTTGTTGAG	ATTO700 at 5´end
Fill-In-550	CCATGATGACTCTTCTGGTC	ATTO550 at 5´end
Fill-In-590	CCATGATGACTCTTCTGGTC	ATTO590 at 5´end
Fill-In-647N	CCATGATGACTCTTCTGGTC	ATTO647N at 5´end
Fill-In-700	CCATGATGACTCTTCTGGTC	ATTO700 at 5´end

C

MspI
HpaII

CTCAACAACTAACTACCAT**CCGG**ACCAGAAGAGTCATCATGG
GAGTTGTTGATTGATGGTA**GGCC**TGGTCTTCTCAGTAGTACC★ 42mer

CGGACCAGAAGAGTCATCATGG
CTGGTCTTCTCAGTAGTACC★ 21mer

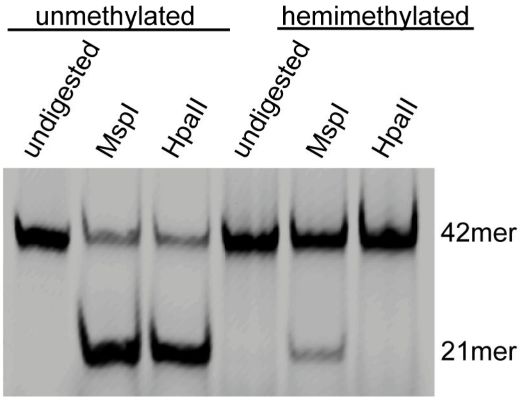

Supplementary Figure S7. Histone-tail peptide and DNA sequences and quality control of DNA substrates. (**A**) Amino acid sequence of TAMRA-labelled peptides for *in vitro* histone-tail peptide binding assays. Histone-tail peptides were purchased as TAMRA conjugates (PSL, Germany). (**B**) DNA oligos used for preparation of double-stranded probes for *in vitro*

DNA binding assays. M: 5-methyl-cytosine. For hybridization, DNA oligos were mixed in equimolar amounts, heated to 92°C and cooled down to room temperature. DNA substrates for Figure 2F were completed in a primer extension reaction. By using a control set of DNA probes with identical sequence but different fluorescent labels we observed effects due to probe preparation and/or unspecific binding of ATTO dyes (data not shown). The values obtained from the control set were used to normalize every probe/protein pair. (**C**) Quality control of DNA substrates. Un- and hemimethylated DNA substrates (2 pmol; Atto647N labelled) were digested with 1 unit MspI or HpaII and analyzed by 15% non-denaturing PAGE for CpG methylation. Note that unmethylated DNA substrate is digested by both enzymes, whereas hemimethylated substrate is only cut by MspI. Enzyme recognition motifs are boxed and asterisks represent ATTO labels.

Supplementary References

Abramoff MD, Magelhaes, P.J., Ram, S.J. 2004. Image Processing with ImageJ. Biophotonics International 11:36-42.

Arnold K, Bordoli L, Kopp J, Schwede T. 2006. The SWISS-MODEL workspace: a web-based environment for protein structure homology modelling. Bioinformatics 22:195-201.

Guex N, Peitsch MC. 1997. SWISS-MODEL and the Swiss-PdbViewer: an environment for comparative protein modeling. Electrophoresis 18:2714-23.

Rothbauer U, Zolghadr K, Muyldermans S, Schepers A, Cardoso MC, Leonhardt H. 2008. A versatile nanotrap for biochemical and functional studies with fluorescent fusion proteins. Mol Cell Proteomics 7:282-9.

Schrodinger, LLC. 2010. The PyMOL Molecular Graphics System, Version 1.3editor^editors.

Sporbert A, Domaing P, Leonhardt H, Cardoso MC. 2005. PCNA acts as a stationary loading platform for transiently interacting Okazaki fragment maturation proteins. Nucleic Acids Res 33:3521-8.
